# Dedicated bacterial esterases reverse lipopolysaccharide ubiquitylation to block immune sensing

**DOI:** 10.21203/rs.3.rs-2986327/v1

**Published:** 2023-07-12

**Authors:** Magdalena Szczesna, Yizhou Huang, Rachel E. Lacoursiere, Francesca Bonini, Vito Pol, Fulya Koc, Beatrice Ward, Paul P. Geurink, Jonathan N. Pruneda, Teresa L.M. Thurston

**Affiliations:** 1 Department of Infectious Disease, Centre for Bacteriology Resistance Biology, Imperial College London, London, SW7 2AZ, UK; 2 Department of Molecular Microbiology & Immunology, Oregon Health & Science University, Portland, OR 97239, USA; 3 Department of Cell and Chemical Biology, Leiden University Medical Center, Leiden, the Netherlands

## Abstract

Pathogenic bacteria have evolved diverse mechanisms to counteract cell-autonomous immunity, which otherwise guards both immune and non-immune cells from the onset of an infection^1,2^. The versatile immunity protein Ring finger protein 213 (RNF213)^3–6^ mediates the non-canonical ester-linked ubiquitylation of lipopolysaccharide (LPS), marking bacteria that sporadically enter the cytosol for destruction by antibacterial autophagy^4^. However, whether cytosol-adapted pathogens are ubiquitylated on their LPS and whether they escape RNF213-mediated immunity, remains unknown. Here we show that *Burkholderia* deubiquitylase (DUB), TssM^7–9^, is a potent esterase that directly reverses the ubiquitylation of LPS. Without TssM, cytosolic *Burkholderia* became coated in polyubiquitin and autophagy receptors in an RNF213-dependent fashion. Whereas the expression of TssM was sufficient to enable the replication of the non-cytosol adapted pathogen *Salmonella*, we demonstrate that *Burkholderia* has evolved a multi-layered defence system to proliferate in the host cell cytosol, including a block in antibacterial autophagy^10–12^. Structural analysis provided insight into the molecular basis of TssM esterase activity, allowing it to be uncoupled from isopeptidase function. TssM homologs conserved in another Gram-negative pathogen also reversed non-canonical LPS ubiquitylation, establishing esterase activity as a bacterial virulence mechanism to subvert host cell-autonomous immunity.

## *RNF213* senses intracellular *Burkholderia*

**Fig. 1:**
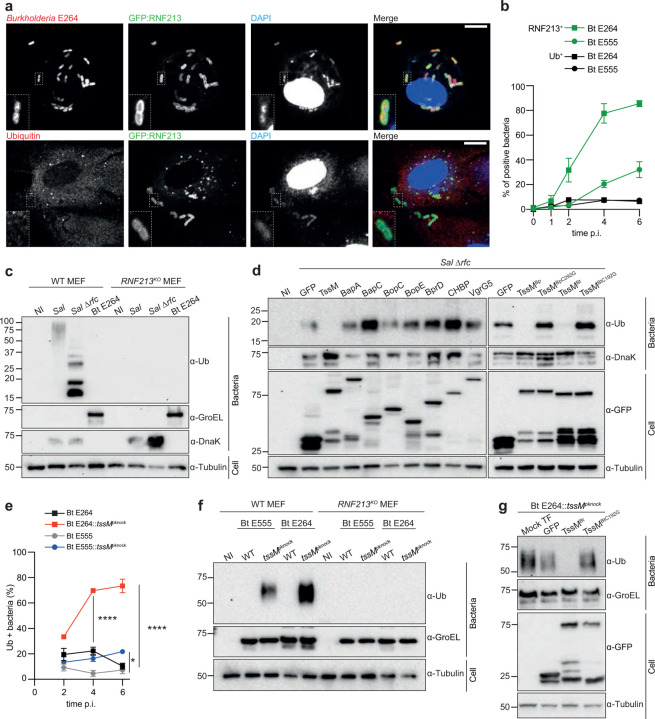
*Burkholderia* effector TssM counteracts the activity of RNF213. **a)** Representative confocal microscopy images of RNF213-knockout (RNF213^KO^) mouse embryonic fibroblasts (MEFs)^4^ stably expressing GFP-RNF213, infected with *B. thailandensis* E264 (bottom panel) or E264 pH4-GroS-RFP (top panel) and fixed at 6 h post-invasion (p.i.). Samples were labelled with an anti-ubiquitin (Ub) antibody when indicated. Scale bar - 10 μm. **b)** Immunofluorescence-based quantification of RNF213-positive *B. thailandensis* E264 and E555 strains in RNF213^KO^ MEFs stably expressing GFP-RNF213, or ubiquitin-positive bacteria following infection of WT MEFs. **c)** Immunoblots of indicated *Salmonella* (*Sal*) and *B. thailandensis* (Bt) strains isolated from infected MEFs at 4 h or 24 h p.i., respectively or non-infected (NI) cells as a control. Cell lysates and isolated intracellular bacteria were immunoblotted with the indicated antibodies. DnaK and GroEL were used as loading controls for *Salmonella* and *Burkholderia*, respectively. **d)** Immunoblot analysis of Δ*rfc Salmonella* isolated from infected HEK293ET cells that were transiently expressing the indicated GFP-tagged effector from *B. pseudomallei* or *B. thailandensis*. **e)** Immunofluorescence-based quantification of the percentage of Ub-coated bacteria over time in WT MEFs infected with indicated *B. thailandensis* strains. **f)** Immunoblot analysis of indicated *B. thailandensis* strains isolated from infected WT or RNF213^KO^ MEFs at 24 h p.i. **g)** Immunoblot analysis of HEK293ET cells transiently transfected with plasmids encoding the indicated GFP-tagged effector and infected with the Bt E264::*tssM*^*pknock*^ strain. **b,e)** Values show mean of three biological repeats ± SEM. Other data are representative of at least three biological repeats. Statistical significance was assessed by two-way ANOVA with Sidak’s multiple comparisons test **(e)**; * P < 0.05; **** < 0.0001.

*Burkholderia pseudomallei* (Bp), which is endemic in large parts of the tropics, causes melioidosis in humans^13^. Species related to *Burkholderia pseudomallei*, including *B. mallei* and *B. thailandensis* (Bt), replicate freely in the host cell cytosol of both non-phagocytic and phagocytic immune cells^14^. Despite indications that *Burkholderia* evade antibacterial autophagy^10,12^, it remains to be determined whether, and how, cytosolic bacteria, including *Burkholderia* spp., evade restriction by the newly identified immune sensor, RNF213, which restricts the growth of numerous intracellular pathogens including bacteria, parasites and viruses^3–6^. Immunofluorescence microscopy showed that *B. thailandensis* strain E264 became coated with RNF213 (Fig. 1a). The percentage of RNF213-coated E264 bacteria accumulated over time, reaching more than 80% at 6 hours post-invasion (Fig. 1b). Up to 30% of a second *B. thailandensis* strain, E555, containing a polysaccharide capsule like that of *B. pseudomallei*^15,16^, was also coated with RNF213 (Fig. 1b). RNF213 is a large and unusual E3 ligase that mediates the direct and non-canonical modification of *Salmonella* LPS with ubiquitin (Ub)^4,17^. However, despite RNF213 recruitment to *B. thailandensis*, less than 10% of either strain accumulated a ubiquitin coat, even at 6 hours post-invasion (Fig. 1a,b). To further investigate this potential *Burkholderia*-mediated block in RNF213 activity, we analysed the ubiquitylation of cytosolic bacteria by immunoblot. Bacteria isolated from infected cells yielded a high molecular weight, ubiquitin-positive smear for wildtype (WT) *Salmonella* that was absent in non-infected cells and dependent on RNF213 (Fig. 1c). As shown previously, the semi-rough LPS mutant *Salmonella* strain (Δ*rfc*), containing just one O-antigen unit, had a distinct, ubiquitin-positive banding pattern of lower molecular mass, indicating that LPS is the target of RNF213-mediated ubiquitylation^4^. However, no ubiquitin smear was detected with *B. thailandensis* obtained from infected WT or *RNF213*^KO^ cells (Fig. 1c). Therefore, RNF213 detects intracellular *B. thailandensis*, but the evident lack of associated ubiquitylation strongly suggests that *Burkholderia* evades, inhibits, or reverses the activity of RNF213.

## *Burkholderia* TssM counters RNF213

We hypothesised that a secreted *Burkholderia* virulence protein (effector) counteracts the activity of RNF213. To test this, we exploited the RNF213-mediated ubiquitylation of Δ*rfc Salmonella* and exogenously expressed eight previously defined and verified *B. pseudomallei* secreted effectors^8,18^ to search, *in trans*, for anti-RNF213 activity. Following infection of cells expressing GFP, or GFP-tagged effectors BapA, BapC, BopC, BopE, BprD, CHBP or VgrG5, Δ*rfc Salmonella* became ubiquitylated. In stark contrast, *Salmonella* ubiquitylation was entirely absent in cells expressing TssM^Bp^, indicating that this protein exhibits *in trans* activity that protects cytosolic *Salmonella* (Fig. 1d).

TssM is a cysteine hydrolase with specific isopeptidase activity towards polyubiquitin chains and an important *in vivo* anti-inflammatory role^7–9^. The protein is highly conserved among *B. pseudomallei* complex species, and the expression of *B. thailandensis* TssM (TssM^Bt^) also ablated the ubiquitylation of Δ*rfc Salmonella* (Fig. 1d). Consistent with an enzymatic role in hydrolysing ubiquitin modifications, the ubiquitin-positive signal remained upon expression of the catalytically inactive variants^9^, TssM^BpC292G^ and TssM^BtC192G^ (Fig. 1d).

To test whether the presence of TssM explains why *B. thailandensis* does not acquire the expected ubiquitin coat, despite recruitment of RNF213, we created E555::*tssM*^*pknock*^ and E264::*tssM*^*pknock*^ mutant strains to prevent its expression. Analysis of infected cells by immunofluorescence microscopy revealed that over 70% of E264::*tssM*^*pknock*^ *B. thailandensis* accumulated a ubiquitin coat, compared to less than 10% of WT bacteria at 6 hours post-invasion (Fig. 1e **and Extended Data Fig. 1**). E555::*tssM*^*pknock*^ bacteria also exhibited a significantly greater percentage of ubiquitin-coating when compared to their isogenic WT strain (Fig. 1e). Furthermore, immunoblotting demonstrated that E555::*tssM*^*pknock*^ and E264::*tssM*^*pknock*^ mutant bacteria, but not WT bacteria, were ubiquitylated inside infected cells and that this required RNF213 (Fig. 1f). The exogenous expression of WT, but not catalytically inactive TssM^BtC192G^, ablated the ubiquitin-positive signal that otherwise accumulated on E264::*tssM*^*pknock*^ bacteria during infection (Fig. 1g). We conclude that RNF213 mediates the ubiquitylation of both capsulated and non-capsulated *B. thailandensis*, but only when the bacteria lack TssM.

## TssM blocks autophagy receptor recruitment

**Fig. 2:**
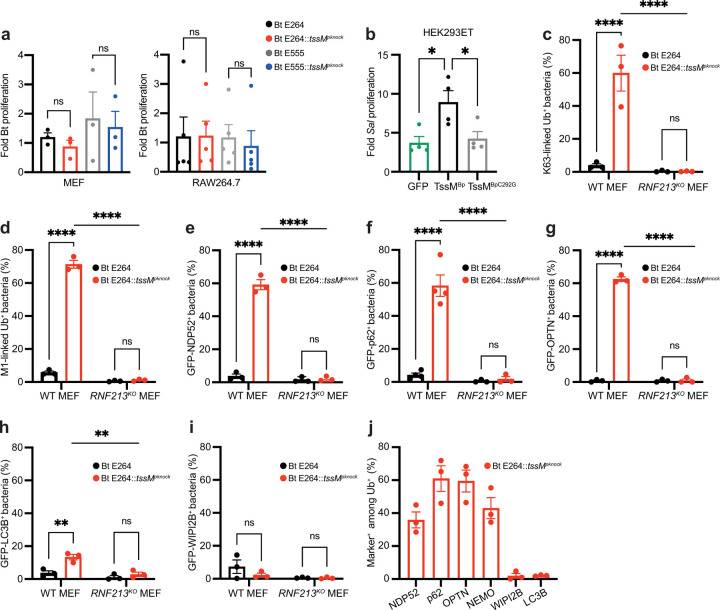
TssM blocks ubiquitin accumulation and autophagy receptor recruitment. Colony forming unit assays were used to determine the fold replication at 6 h p.i. of **a)** the indicated *Burkholderia* strains in MEFs or RAW264.7 macrophages, and **b)** of *Salmonella* in HEK293ET cells expressing the indicated GFP-tagged protein. Quantification of the percentage of **c)** K63-linked or **d)** M1-linked ubiquitin-positive bacteria. Quantification of the percentage of *B. thailandensis* colocalising with **e)** GFP-NDP52, **f)** GFP-p62, **g)** GFP-OPTN, **h)** GFP-LC3B and **i)** GFP-WIPI2B. Percentages of marker positive bacteria were determined by microscopy in WT and RNF213^KO^ MEFs at 6 h p.i. **j)** Percentage of marker-positive *B. thailandensis* among ubiquitin-coated bacteria in WT MEFs. Data represent the mean and SEM of at least three independent biological repeats. Statistical significance was assessed by two-way ANOVA with Tukey’s multiple comparisons test **(c-j)** or one-way ANOVA **(a-b)**; * P < 0.05; *** < 0.001.

The RNF213-mediated ubiquitylation of *Salmonella* LPS^4^, GarD-deficient *Chlamydia-*containing inclusions^3^ and *Toxoplasma*-containing vacuoles^6^ functions as a ubiquitin-dependent “eat-me” signal^2,19^ that induces antimicrobial autophagy and pathogen elimination. However, the intracellular bacterial burden between WT and *tssM*-mutant *Burkholderia* was indistinguishable in both MEFs and RAW264.7 macrophages (Fig. 2a). This finding, which is in line with previous reports for the *tssM* mutant of *B. mallei*^9^, suggests that *Burkholderia* have additional virulence mechanisms that counteract ubiquitin-mediated cell-autonomous immunity. This prompted us to test whether TssM could promote the replication of the non-cytosol adapted pathogen, *Salmonella*. Exogenous expression of WT TssM^Bp^, but not catalytically inactive TssM^BpC292G^, was sufficient to promote *Salmonella* replication (Fig. 2b), providing further evidence that *Burkholderia* has at least one additional mechanism to block host-mediated restriction of cytosolic bacteria.

To investigate why E264::*tssM*^*pknock*^ bacteria, which become decorated with the ubiquitin “eat-me” signal (Fig. 1e,f), replicate as efficiently as WT *Burkholderia*, we next analysed the associated polyubiquitin signals. At 6 hours post-invasion, the majority of ubiquitin-coated E264::*tssM*^*pknock*^ and E555::*tssM*^*pknock*^ bacteria (identified with an antibody that detects diverse ubiquitin chain types and mono-ubiquitin) accumulated K63- and linear M1-linked polyubiquitin chains, both of which are important signals for antibacterial autophagy^20,21^ (**Extended Data Fig. 2a**). Approximately 60% of all intracellular E264::*tssM*^*pknock*^ bacteria were coated with M1- or K63-linked polyubiquitin, compared to less than 5% of WT E264 bacteria, and this was dependent on RNF213 (Fig. 2c,d).

As these polyubiquitin signals initiate the recruitment of ubiquitin-binding autophagy receptors Optineurin (OPTN)^22^, NDP52^23^ and p62^24^, we next explored the recruitment of these proteins to *Burkholderia*. Consistent with the accumulation of polyubiquitin signals, NDP52, p62 and OPTN were recruited to approximately 60% of E264::*tssM*^*pknock*^ bacteria in an RNF213-dependent manner, in contrast to fewer than 5% of WT bacteria (Fig. 2e–g). In line with the overall lower levels of ubiquitin coating on E555::*tssM*^*pknock*^ bacteria (Fig. 1e), whereas the percentage of marker-positive bacteria in this strain was elevated compared to WT E555 bacteria in an RNF213-dependent manner, it remained below 20% of total bacteria (**Extended Data Fig. 2b-d**). This finding is also consistent with our previous observation that RNF213 was recruited to a lower percentage of WT E555 *B. thailandensis* (Fig. 1b). Overall, we find that without TssM, *B. thailandensis* becomes coated with polyubiquitin and associated with autophagy receptors.

## Additional virulence mechanisms block antibacterial autophagy

The ubiquitin-dependent recruitment of autophagy receptors enables delivery of the marked “cargo” to an LC3-positive, double membrane autophagosome^22–25^. Despite our finding that individual autophagy receptors coated approximately 60% of E264::*tssM*^*pknock*^ bacteria, only 15% of these mutant bacteria were positive for LC3 in MEFs (Fig. 2h) and less than 10% in RAW264.7 macrophages (**Extended Data Fig. 2e**). To investigate this further, we analysed recruitment of WIPI2B, which is required for LC3 conjugation and autophagy of *Salmonella*^26^. The percentages of WIPI2B-positive E264::*tssM*^*pknock*^ and WT E264 were low and indistinguishable at 6 hours post-invasion (Fig. 2i). When marker-positive bacteria were quantified among the fraction of ubiquitin-positive E264::*tssM*^*pknock*^ bacteria, the failure to recruit critical autophagy proteins WIPI2B and LC3B was evident (Fig. 2j). Similarly, whether quantified among the total or the ubiquitin-positive population, it was evident that WIPI2B and LC3B were associated with fewer E555::*tssM*^*pknock*^ bacteria than the autophagy receptors (**Extended Data Fig. 2f-h**). Together, this suggests that the failure in antibacterial autophagy occurs due to a block in its initiation. As *B. pseudomallei* prevents LC3 lipidation and association of LC3 with bacteria through the action of BopA^10,12^, we propose that this, or indeed other additional mechanisms that also include the polysaccharide capsule when present, cooperate with the anti-RNF213 activity of TssM to promote the intracellular replication of *Burkholderia* spp.

## LPS represents the ubiquitylated substrate

**Fig. 3:**
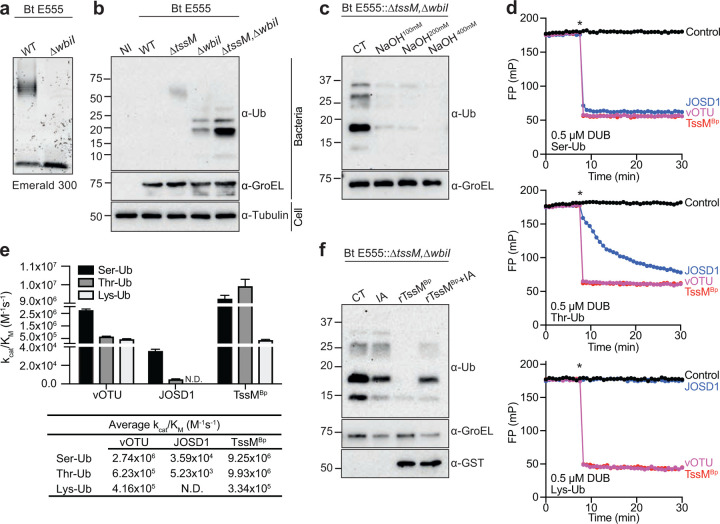
TssM is a ubiquitin esterase that hydrolyses ubiquitylated LPS. **a)** Emerald 300 stain of LPS from indicated *B. thailandensis* strains grown in LB. **b)** Immunoblot analysis of *B. thailandensis* strains isolated from infected MEFs, 24 h p.i. **c)** Bacteria isolated from MEFs infected with the E555::Δ*wbiI*,Δ*tssM* strain were lysed and incubated with 100, 200 or 400 mM NaOH for 30 min prior to immunoblot analysis. **d)** Representative FP data monitoring cleavage of Rho-S(Ub)G (Ser-Ub), Rho-T(Ub)G (Thr-Ub), and Tamra-K(Ub)G (Lys-Ub) substrates following addition of the DUB (indicated by an asterisk). **e)** Catalytic efficiencies (mean + SEM of three repeats) for all enzyme-substrate combinations, with the exception of JOSD1 which had no detectable isopeptidase activity. **f)** Bacteria isolated from MEFs infected with the E555::Δ*wbiI*,Δ*tssM* strain were incubated with recombinant His-GST-tagged TssM^BpΔN191^ (rTssM^Bp^) +/− iodoacetamide (IA) for 30 min prior to immunoblot analysis. **a-c)** and **e)** representative of three biological repeats.

Current data strongly suggest that RNF213 ubiquitylates *Salmonella* LPS, rather than a proteinaceous substrate^4^, but whether this is the case for a host cytosol-adapted bacteria has not been tested. We therefore hypothesised that the RNF213-dependent ubiquitylation of *tssM*^*pknock*^ bacteria also represented modification of bacterial LPS. To test this, we created an E555::Δ*wbiI* mutant or an E555::Δ*tssM*,Δ*wbiI* double mutant, in which the lack of WbiI prevents formation of long O-antigen, leaving only the lipid A and inner core of the LPS moiety (Fig. 3a). Surprisingly, a defined, lower molecular weight ubiquitin-positive banding pattern was detected in LPS-enriched lysates from the E555::Δ*wbiI*-infected cells (Fig. 3b). This suggested that deleting the O-antigen removes a physical barrier to the ubiquitylation of *B. thailandensis*, perhaps by providing better access to the lipid A moiety. This ubiquitin-positive banding pattern resembled that detected for the Δ*rfc* mutant of *Salmonella* (Fig. 1c) and importantly, ubiquitylation of the E555::Δ*tssM*,Δ*wbiI* double mutant was enhanced compared to the E555::Δ*wbiI* mutant (Fig. 3b).

The strict correlation between the molecular weight of the detected ubiquitin signal and O-antigen size strongly suggests the direct ubiquitylation of *B. thailandensis* LPS. As the LPS core lacks amino groups suitable for amide-linked ubiquitylation^4^, it is likely that available hydroxyl groups are modified^27^, creating an ester-linked ubiquitin. To test this, we treated E555::Δ*tssM*,Δ*wbiI* bacteria isolated from infected cells with sodium hydroxide, which selectively hydrolyses ester-linked conjugates. The addition of increasing concentrations of sodium hydroxide clearly resulted in progressive loss of the ubiquitin signature (Fig. 3c). Therefore, we conclude that without TssM, *Burkholderia* undergo RNF213-mediated non-canonical ubiquitylation of LPS.

## TssM is a highly potent ubiquitin esterase

TssM has reported isopeptidase activity^9^, cleaving amide bonds within a polyubiquitin chain, yet our data imply that TssM reverses ester-linked ubiquitylation. To test whether TssM exhibits both isopeptidase and esterase activity, as seen for several other members of the ubiquitin-specific protease (USP) family of DUBs^28^, we purified recombinant His-GST-tagged TssM^BpΔN191^. This variant lacks the first 191 amino acids but contains the intact catalytic domain^9^, and was active against a Ub-Propargylamide (Ub-PA)^29^ activity-based probe (**Extended Data Fig. 3a**). As models for the analysis of esterase activity, we synthesised substrates containing ubiquitin linked to the hydroxyl group of Serine (Ser) or Threonine (Thr), as well as the isopeptide-linked Tamra-K(Ub)G (Lys-Ub)^30^ as a control substrate. Cleavage of these substrates was monitored by a decrease in fluorescence polarisation (FP) following release of the fluorescent Ser/Thr/Lys-containing peptide from ubiquitin. As expected, the human ester-specific DUB JOSD1^28^ preferred the Rho-S(Ub)G and Rho-T(Ub)G substrates (hereafter referred to as Ser-Ub and Thr-Ub) over isopeptide-linked Lys-Ub, whereas the Crimean-Congo Haemorrhagic Fever Virus DUB, vOTU^31^, cleaved all ubiquitin modifications indiscriminately (Fig. 3d, **Extended Data Fig. 3b,c**). TssM cleaved both ester- and isopeptide-linked ubiquitin substrates very efficiently, even at low or subnanomolar enzyme concentrations (Fig. 3d, **Extended Data Fig. 3d**). In fact, TssM exhibited extremely robust ubiquitin esterase activity, with catalytic efficiencies approaching 1 × 10^7^ M^−1^s^−1^ against the Ser- and Thr-linked ubiquitin substrates (Fig. 3e). Compared to the Lys-Ub substrate, TssM was nearly 30-fold more active toward ester-linked ubiquitin, whereas the control enzyme vOTU showed just a four-fold preference. Remarkably, TssM demonstrated up to 1900-fold more activity toward ester-linked Ub than JOSD1, making it a highly potent ubiquitin esterase. This provides direct evidence that TssM exhibits both isopeptidase and esterase activity.

Given the esterase activity of TssM, we tested whether recombinant TssM^BpΔN191^ directly removed ubiquitin from bacterial LPS. Treatment of E555::Δ*tssM*,Δ*wbiI* lysates obtained from infected cells with TssM^BpΔN191^ removed the ubiquitin signal otherwise detected in control conditions, or when iodoacetamide (an established inhibitor of cysteine-dependent DUBs) was included in the reaction (Fig. 3f). Similar results were obtained when Δ*rfc Salmonella* lysates from infected cells were treated with TssM^BpΔN191^ (**Extended Data Fig. 3e**). We conclude that TssM enzymatically hydrolysed ester-linked ubiquitylated LPS from both *Burkholderia* and *Salmonella*, providing the first physiological characterisation of a ubiquitin esterase.

## Molecular basis for TssM activity

**Fig. 4:**
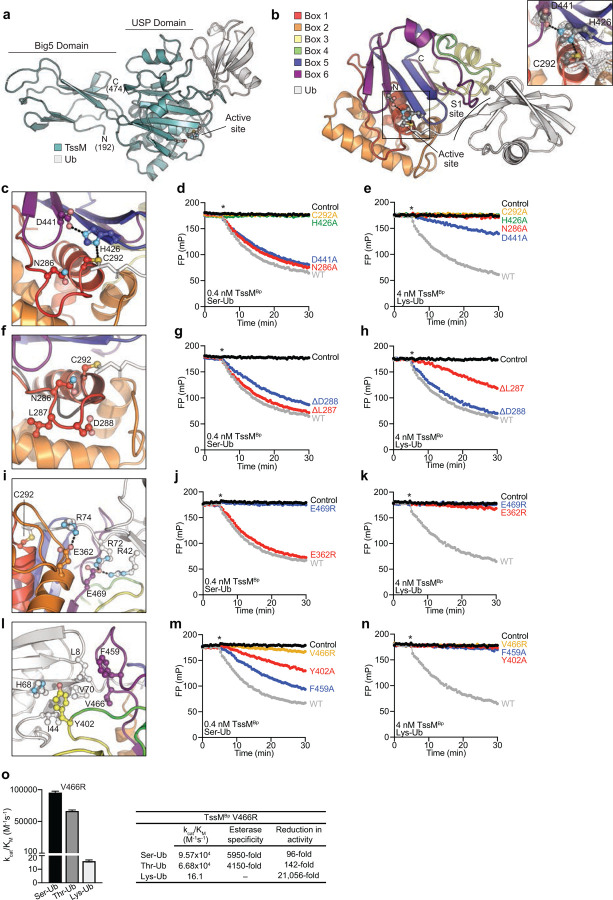
Structural basis of TssM^Bp^ esterase activity. **a)** Crystal structure of TssM^BpΔN191^ (teal) bound to Ub (grey). **b)** Close-up of the TssM USP domain coloured by box regions 1–6, with Ub (grey) shown in the S1 site. Representative 2|Fo|-|Fc| electron density is shown at 1σ. **c)** TssM catalytic triad C292, H426, and D441, as well as the oxyanion hole N286 are shown. Hydrogen bonds are shown as dashed lines. Effects of mutation on TssM activity are shown for **d)** Ser-Ub and **e)** Lys-Ub substrates. **f)** The extended TssM Cys-loop (red) in comparison to a typical USP Cys-loop (grey). Activity data for TssM truncations ΔL287 and ΔD288 are shown for **g)** Ser-Ub and **h)** Lys-Ub substrates. **i)** Coordination of the Ub C-terminus and R42 by TssM E362 and E469. Activity data of E362R and E469R mutants are shown for **j)** Ser-Ub and **k)** Lys-Ub substrates. **l)** Coordination of the Ub Ile44 hydrophobic patch by TssM S1 site residues Y402, F459, and V466 is shown. The effects of mutations on cleavage of **m)** Ser-Ub and **n)** Lys-Ub are shown. In all panels, DUB addition is indicated by an asterisk. WT and control data in panels d and g, e and h, j and m, and k and n are identical and reproduced for clarity as data were collected in the same experiment. **o)** Rate constants and correlating catalytic efficiency (k_cat_/K_M_) derived for esterase-specific TssM^BpΔN191^ V466R are shown as mean + SEM. All FP data are representative of three biological repeats.

We determined a 2.5 Å crystal structure of TssM^BpΔN191^ covalently bound to Ub at its active site to visualise the molecular basis for ubiquitin esterase activity (Fig. 4a, **Extended Data Fig. 4a-c**, Extended Data Table 1). The structure of TssM revealed a Big5 (bacterial Ig-like domain 5) fold N-terminal to a catalytic USP-type DUB module (Fig. 4a). Ig-like domains facilitate protein or ligand interactions^32^, and we detected unexplained electron density in the b1 groove, indicating the possibility of a co-purified ligand from expression in *E. coli* (**Extended Data Fig. 4d,e**). However, when TssM was overexpressed in cells the Big5 domain was not required for deubiquitylation of LPS (**Extended Data Fig. 4f**). USP domains are typically composed of six conserved “box” regions that can be interrupted by sequence insertions of varying lengths^33^. The TssM USP domain is very minimal and contains no sequence insertions. Boxes 1, 2, 5, and 6 are well-conserved and form the core USP module, including the catalytic triad (Fig. 4b, **Extended Data Fig. 4g-i**). Ubiquitin is typically bound at the S1 substrate-binding site by a set of “fingers” encoded within Boxes 3 and 4, as well as a Box 4 “blocking loop” that guides the ubiquitin C-terminus into the active site. In contrast, TssM has no recognizable finger structure and compensates for an extremely short Box 4 region by encoding an analogous blocking loop within Box 6 instead (Fig. 4b, **Extended Data Fig. 4g-i**).

Within the TssM active site lies an aligned Cys-His-Asp catalytic triad, as well as a conserved Asn that forms the oxyanion hole (Fig. 4c). Mutation of these sites ablated or diminished isopeptidase activity toward a Lys-Ub substrate, but interestingly the acidic Asp or oxyanion hole Asn were not required for ubiquitin esterase activity (Fig. 4d,e, **Extended Data Fig. 5a,b**). Among USPs the length of the so-called Cys-loop that precedes that catalytic Cys is almost perfectly conserved. TssM encodes a one amino acid insertion within the Cys-loop (Fig. 4f), but its truncation had a minimal effect on esterase activity and instead ΔL287 specifically reduced isopeptidase function (Fig. 4g,h, **Extended Data Fig. 5a,c**). As the ubiquitin C-terminus threads into the active site, TssM coordinates the basic R42, R72, and R74 residues of ubiquitin with acidic residues E362 and E469 in Boxes 2 and 6, respectively (Fig. 4i). Mutation of either residue severely diminished isopeptidase activity, but esterase activity was more significantly impacted by an inability to coordinate R42 and R72 with residue E469 (Fig. 4j,k, **Extended Data Fig. 5a,d**). Lastly, the remainder of the S1 site is composed of hydrophobic interactions to the Ile44 hydrophobic patch of ubiquitin stemming from TssM Boxes 3 and 6 (Fig. 4l). Mutations in these hydrophobic TssM residues (Y402, F459, or V466) reduced esterase activity to varying extents, and ablated isopeptidase function (Fig. 4m,n, **Extended Data Fig. 5a,e**).

As we noted that several of our structure-guided mutations in the TssM active site or S1 site affected isopeptidase function more so than esterase, we sought to determine if any had completely lost isopeptidase activity. We selected N286A, E362R, F459A, V466R, and E469R as TssM mutants that more significantly impacted isopeptidase activity (Fig. 4c–n, **Extended Data Fig. 5a-e**). At higher enzyme concentrations, all mutants tested were able to cleave the Lys-Ub substrate efficiently except V466R and E469R, which were severely impaired (**Extended Data Fig. 5f**). We selected TssM V466R for further characterization, as it retained more esterase activity compared to E469R. Despite a ~100-fold reduction in the k_cat_/K_M_ for Ser-Ub and Thr-Ub substrates, the V466R mutant exhibited a ~21,000-fold reduction in k_cat_/K_M_ for Lys-Ub, amounting to an ~5,000-fold specificity for ester- over isopeptide-linked substrates (Fig. 4o, **Extended Data Fig. 5g**). We suggest that TssM esterase activity is more compatible with weak or transient substrate encounters, with the differential dependency on certain active site features allowing us to uncouple esterase and isopeptidase activity.

## Conservation of ubiquitin esterase activity

**Fig. 5:**
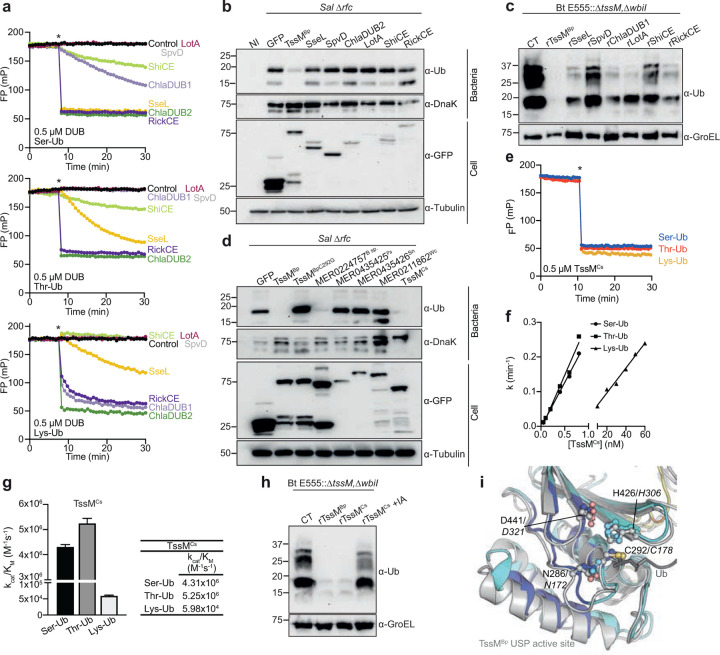
Esterase activity in other bacterial peptidases. **a)** Representative FP data monitoring cleavage of Ser-Ub, Thr-Ub, and Lys-Ub substrates following addition of the DUB (indicated by an asterisk). **b)** Immunoblot analysis of Δ*rfc Salmonella* isolated at 6 h p.i. from infected HEK293ET cells transiently expressing the indicated GFP-tagged bacterial cysteine hydrolases. The panel includes SseL and SpvD from *Salmonella*^43,44^, ChlaDUB1 and ChlaDUB2 from *Chlamydia*^44^ as well as LotA from *Legionella*^45^, ShiCE from *Shigella* and RickCE from *Rickettsia*^44^. **c)** Bt E555::Δ*tssM*,Δ*wbiI* mutant bacteria isolated from infected MEF cells were treated with 0.5 μM of the indicated purified bacterial DUB. **d)** Immunoblot analysis of Δ*rfc Salmonella* isolated from infected cells expressing the indicated GFP-tagged putative C19 peptidases of *Burkholderia* (*B sp*.), *Parachlamydia acanthamoebae* (*Pa*), *Simkania negevensis* (*Sn*)*, Waddlia chondrophila* (*Wc*) and *Chromobacterium sinusclupearum* (*Cs*). **e)** Representative FP data monitoring cleavage of Ser-Ub, Thr-Ub, and Lys-Ub substrates with 0.5 μM TssM^Cs^. Data were collected in triplicate and **f)** rate constants and **g)** catalytic efficiency of the TssM^Cs^ protein towards each substrate are shown as mean + SEM. **h)** Immunoblot analysis of E555::Δ*tssM*,Δ*wbiI B. thailandensis* isolated from infected MEFs and incubated with control (CT), recombinant His-GST-tagged TssM^BpΔN191^ (rTssM^Bp^) or TssM^Cs^ +/− iodoacetamide (IA) for 30 min. **i)** Structural alignment of the active site of TssM (grey) and the AlphaFold model of TssM^Cs^ (coloured) showing similarity between the two proteins surrounding the active site. Catalytic residues for TssM are labelled, with aligned residues in TssM^Cs^ labelled in italics. **c,h)** rTssM^Bp^ refers to recombinant His-GST-tagged TssM^BpΔN191^.

Next, we asked how common esterase activity is among an array of bacterial DUBs. As observed with human DUBs^28^, many bacterial DUBs were capable of ubiquitin esterase activity at high (0.5 μM) enzyme concentration (Fig. 5a). Despite this, only TssM protected *Salmonella* from LPS ubiquitylation when each DUB was expressed in *trans* (Fig. 5b). Furthermore, recombinant TssM was the most potent DUB tested, removing all anti-ubiquitin detected bands from E555::Δ*tssM*,Δ*wbiI* bacteria isolated from infected cells (Fig. 5c). This revealed a specificity within TssM, compared to several other bacterial DUBs, for the removal of ubiquitin from LPS.

To extend our analysis of substrate specificity, we specifically examined a subset of bacterial peptidases from the C19 family, for which TssM is a member. Using MEROPS, we selected C19 peptidases from intracellular bacteria including other *Burkholderia* species, *Parachlamydia acanthamoebae*^34^, *Simkania negevensis*^35^, and *Waddlia chondrophila*^36^ and tested whether they could cleave ubiquitylated LPS when expressed *in trans*. Of these, only peptidases from *Burkholderia* blocked the ubiquitylation of *Salmonella* LPS (Fig. 5d). We then used NCBI BLAST against the *B. pseudomallei* TssM and identified an additional putative orthologue conserved in several species of *Chromobacterium* (**Extended Data Fig. 6a**), a Gram-negative bacterium associated with rare opportunistic infections that involve growth in the host cell cytosol^37–39^. Surprisingly, despite only 33% sequence conservation with TssM^Bp^, expression of the *Chromobacterium* orthologue, denoted as TssM^Cs^, protected *Salmonella* from host-mediated ubiquitylation, indicative of esterase activity (Fig. 5d). Recombinant TssM^Cs^ protein was active on all fluorescent substrates tested (Fig. 5e, **Extended Data Fig. 6b**), and exhibited over 100-fold greater activity toward the ester-linked substrates than the isopeptide substrate (Fig. 5f,g). Finally, as recombinant TssM^Cs^ hydrolysed ubiquitin from the LPS of E555::Δ*tssM*,Δ*wbiI* (Fig. 5h), we compared the AlphaFold model^40,41^ of TssM^Cs^ to our TssM structure to assess structural homology (Fig. 5i, **Extended Data Fig. 6c,d**). Consistent with similar activities toward ubiquitylated LPS, there was considerable alignment between the active site regions, where the AlphaFold model confidence was highest. Together, these findings suggest that ubiquitin esterase activity is encoded by at least two cytosolic bacteria as a mechanism of countering RNF213 defences.

Our finding that some conserved bacterial DUBs are esterases that selectively reverse the ubiquitylation of a non-canonical substrate reveals a previously undescribed molecular mechanism for the evasion of RNF213-mediated cell-autonomous immunity. For cytosolic *Burkholderia* spp., we propose that at least three mechanisms cooperate to counteract RNF213. First, the capsule provides a physical barrier as evidenced by lower RNF213 association with E555 bacteria compared to E264 bacteria, consistent with acapsular *B. pseudomallei* exhibiting a significant reduction in virulence^42^. Second, TssM directly reverses RNF213-mediated LPS ubiquitylation. Lastly, the initiation of autophagy is blocked in a TssM-independent manner, which is supported by the observation that *bopA*-deficient *Burkholderia* accumulate LC3^10^. The multiple layers of protection suggest that overcoming this mechanism of host immunity is of the utmost importance for replication in the cytosol (**Extended Data Fig. 7**). Our work provides the first description of biologically relevant ubiquitin esterase activity and reveals a previously unappreciated virulence mechanism. Furthermore, it raises the possibility that other enzymes with esterase activity mediate the regulation of non-canonical and/or non-proteinaceous ubiquitylated substrates, in disease and beyond. To this end, our highly specific TssM esterase variant provides a valuable tool for future studies that investigate non-canonical ubiquitylation.

## Methods

### Plasmids and cloning

ptCMV plasmids were used for transient expression in mammalian cells. M6P plasmids were used to produce recombinant murine leukemia virus (MLV) for stable expression in mammalian cells^46^. Either pETM30 or pOPINB was used for protein expression in *E. coli*. C19 peptidases, with MEROPS identifiers MER0224757, MER0435425, MER0435426 and MER0211862 were gene synthesised (Invitrogen GeneArt Strings DNA Fragments). The *Burkholderia pseudomallei* genes for Uniprot proteins, Q63K38 (BapA), Q63K40 (BapC), Q63K50 (BopC), Q63K41 (BopE), Q63K45 (BprD), Q63KH5 (CHBP), Q63MX4 (VgrG5) and Q63K53 (TssM) were amplified from K96243 gDNA, provided by Dr Jo Stevens. Mutations and gene truncations were generated by polymerase chain reaction and products were confirmed by DNA sequencing.

### Antibodies

Primary antibodies for immunoblotting: mouse anti-Ub (FK2; BML-PW8810 and UBCJ2; AB_2935893, Enzo Life Science), mouse anti-DnaK (8E2/2, ADI-SPA- 880, Enzo Life Science), goat anti-GroEL (ABIN6292975, antibodies-online), mouse anti-tubulin (E7, DSHB), rabbit anti-GST (G7781, Sigma) and rat anti-GFP (3H9, Proteintech).

Primary antibodies used for immunofluorescence microscopy: α-Ub (1:400, FK2; BML-PW8810 and UBCJ2; AB_2935893, Enzo Life Science) α-M1 (1:400, 1E3, ZRB2114, Merck) α-K63 (1:400, Apu3, 05–1308, Millipore) and α-LC3 (1:300, CTB-LC3–2-IC, Cosmo Bio,). Anti-*Burkholderia* antibody was provided by the United States Army Medical Research Institute of Infectious Diseases, an agency of the U.S. Government (“USAMRIID”).

Secondary antibodies used: Thermo Fisher Scientific (1:500, Alexa-conjugated anti-mouse, anti-goat and anti-rabbit antisera) and Dabco (1:5000, HRP-conjugated reagents).

### Cell culture

HEK293ET, MEFs, RNF213^KO^ MEFs, RNF213^KO^ expressing GFP-RNF213 (all provided by Felix Randow) and RAW264.7 macrophages (ATCC) were maintained in Dulbecco’s modified Eagle’s medium (DMEM; Sigma) supplemented with 10% fetal calf serum (FCS; GIBCO, Life Technologies) at 37 °C in 5% CO_2_. In 24 well-plates, cells were transfected using Lipofectamine 2000 (Life Technologies, Inc.) as per the manufacturer’s instructions. HEK293ET seeded in 10 cm dishes, were transfected using calcium phosphate two days prior infection. Stable cell lines were generated by retroviral transduction with M6P-derived plasmids encoding GFP-NDP52, GFP-p62, GFP-OPTN, GFP-LC3B and GFP-WIPI2B. 48 h post transduction, cells were either selected in blasticidin (5 μg/mL) or sorted by flow cytometry (GFP+ cells).

### Bacterial strains

*Escherichia coli* strain DH5α was used for cloning (Thermo Fisher), and BL21 (DE3) (New England) and Rosetta (DE3) (Millipore) for protein expression. *E. coli CC118* λpir and E. coli S17–1 λpir were used for construction of λpir-dependent vectors and conjugal transfer respectively.

*Salmonella enterica* serovar Typhimurium strain NCTC 12023 was used with Δ*rfc* provided by Dr Felix Randow.

*B. thailandensis* acapsular E264 and capsulated E555, as well as associated fluorescent RFP strains (carrying pHR4-GroS-RFP), were provided by the Defence Science and Technology Laboratory of Porton Down.

To generate mutants in *B. thailandensis*, two different approaches were used. For gene disruption by targeted insertion of a pknock plasmid into the chromosome, an internal fragment of TssM (700–800bp) was amplified by PCR from B*. thailandensis* DNA using primers with engineered NotI and SalI restriction sites. The TssM-pKnock plasmid was introduced into *B*. *thailandensis* E264 or E555 strains via conjugation from *E*. *coli* S17–1 λ*pir*. Conjugants were selected on LB agar with kanamycin/gentamicin and pknock integration in the correct locus was verified by colony PCR.

In-frame deletions of TssM and WbiI were created using the pDM4 suicide vector. A 1 kb fragment containing 500 bp upstream and downstream of the gene of interest was generated by overlap PCR and ligated into pDM4 via its XbaI and SpeI sites. The pDM4 vectors were further modified to insert a I-SceI recognition site that allows generation of a site-specific DNA double-strand break by the I-SceI endonuclease^47^ and sequence verified using pDM4-F and pDM4-R primers. The generated pDM4 plasmids were introduced into *B*. *thailandensis* via conjugation from *E*. *coli* S17–1 λ*pir* and colonies selected on Luria broth (LB) agar with chloramphenicol and gentamicin. To increase the probability of a second recombination event, the pDAI-SceI-SacB plasmid^47^ was transferred by conjugation into the single crossover mutants. Double crossover (chloramphenicol-sensitive colonies) mutants were obtained upon selection with tetracycline and gentamicin. The genotype of the mutants was confirmed by PCR, after which bacteria were grown on salt-free LB agar containing 10% (wt/vol) sucrose for pDAI-SceI-SacB plasmid counter-selection.

The following primers were used:

pKnock-E264-*tssM*-F (cgcggggcggccgcaacgccgcaatcgcacatc)
pKnock-E555-*tssM*-F (cgcggggcggccgcaacgctgcaatcgcacatc)
pKnock-*tssM*-R (cgcggggtcgacttgtagtcgaacgcgacgag)
pDM4-E555-*tssM-*Up-F (cgcgggtctagaacgcccggcgatttcccg)
pDM4-E555-*tssM-*Up-R(ggcgacgcggcgcacgcgcaggcaaggcggaaaaaggct)
pDM4-E555-*tssM-*Down-F(cagcctttttccgccttgcctgcgcgtgcgccgcgtcgcc)
pDM4-E555-*tssM-*Down-R (cgcgggactagtggcccggcgctgtcgtccg)
pDM4-E555-*tssM-*I-sceI-R *(cgcgggactagtattaccctgttatccctaggcccggcgctgtcgtccg)*
pDM4-E555-*wbiI-*Up-F (cgcgggtctagacatcggattgggttgggc)
pDM4-E555-*wbiI-*Up-R (cttgtgccttccttgtagcgattgcgtttattttgatgtg)
pDM4-E555-*wbiI-*Down-F(cacatcaaaataaacgcaatcgctacaaggaaggcacaag)
pDM4-E555-*wbiI-*Down-R (cgcgggactagttcccggacggcgtgcacc)
pDM4-E555-*wbiI-*I-sceI-R *(cgcgggactagtattaccctgttatccctatcccggacggcgtgcacc)*
pDM4-F (acggttgtggacaacaagccagg)
pDM4-R (gtgtttttgaggtgctccag)

### *Salmonella* infections

*S.* Typhimurium strains were grown overnight in LB and subcultured (1:33) in fresh LB for 3.5 h prior to infection at 37 °C.

For analysis of extracted intracellular bacteria, HEK293ET or MEF cells were seeded in a 10 cm dish and infected with 1 mL of bacterial subculture for 15 min at 37 °C. After two or three PBS washes, cells were incubated in 100 μg/mL gentamicin for 1 h, after which the medium was changed to 20 μg/mL gentamicin for the rest of the infection. To enumerate intracellular *Salmonella* by colony forming unit assay, cells in a 12-well plate were infected with 14 μl of undiluted subculture for 15 min at 37 °C. After two PBS washes, the cells were incubated in DMEM supplemented with 10% FCS and 100 μg/mL gentamicin for 2 h, prior to incubation in 20 μg/mL gentamicin. At the desired time point post invasion, cells from triplicate wells were lysed in 1 mL cold PBS with 0.1% Triton X-100 and serial dilutions plated in duplicate on LB agar.

### *B. thailandensis* infection

*Burkholderia* strains were grown overnight in LB and subcultured (1:20) in fresh LB for 3.5 h (optical density of 1.8 to 2 at 600 nm) prior to infection at 37 °C.

For analysis of extracted intracellular bacteria, cells seeded in a 10 cm dish were infected with 100 μl of bacterial subculture. Following centrifugation at 800 × *g* for 5 min, cells were incubated for 2 h at 37 °C to allow bacterial invasion. The cells were then washed two or three times with warm PBS and maintained in fresh media containing 100 μg/mL imipenem for the remaining time of infection.

For the enumeration of colony forming units, MEFs were infected with subcultured bacteria at an MOI of 100 for 2 h and RAW264.7 macrophages infected at an MOI of 10 with an overnight culture for 1 h at 37 °C following centrifugation at 800 × *g* for 5 min. After three PBS washes, cells were maintained in DMEM supplemented with 10% FCS and 100 μg/mL imipenem for the duration of the experiment. Cells from triplicate wells were lysed with 1 mL cold PBS containing 0.1% Triton X-100 and plated in duplicate onto LB agar.

### Bacterial isolation prior to immunoblot analysis

To analyse ubiquitylation of bacteria as previously described^4^, infected cells, at 4 h post-invasion for *Salmonella* and 24 h post-invasion for *Burkholderia*, were lysed in 5 mL of cold lysis buffer (1% Triton X-100, 30 mM Hepes (pH 7.5), 100 mM NaCl, 10 mM MgCl_2_, 10 mM Iodoacetamide and complete protease inhibitor cocktail (Roche)) for 5 min. After a sample was collected for analysis by immunoblot with anti-tubulin (or anti-GFP antibodies when required), lysates were centrifuged at 300 × *g* for 5 min. The bacteria-containing supernatant was collected and centrifuged at 16,100 × *g* for 10 min at 4 °C. The bacterial pellets were washed with lysis buffer and lysed in 50 μl of BugBuster (Merck) supplemented with 10 mM Iodoacetamide and protease inhibitor cocktail. After 5 min of lysis at room temperature, bacterial lysates were centrifuged at 16,100 × *g*, and the supernatant was either directly mixed with Laemmli buffer (for Dnak and GroEL immunoblots) or heat-cleared (90 °C for 15 min) and centrifuged at 16,100 × *g* for 10 min to further purify ubiquitylated LPS prior to analysis by immunoblot with an anti-ubiquitin antibody.

### SDS-PAGE and immunoblotting

Samples, prepared in a Laemmli buffer containing 5% β-mercaptoethanol, were boiled for 5 min prior to protein separation by SDS–PAGE using either 10% or 15% Tris polyacrylamide gels. Following transfer to PVDF membrane (Millipore) and blocking overnight in 5% milk (or BSA) in PBS-Tween, the membranes were incubated with the indicated primary antibodies. HRP-conjugated secondary antibodies (Dako) were used for detection using ECL detection reagents (Cytivia ECL and Pierce ECL2) on a Chemidoc^™^ Touch Imaging System (Bio-Rad).

### Immunofluorescence microscopy

Cells were seeded on coverslips one day prior to infection at a density of 5×10^4^ or 1×10^5^ cells/well. For GFP-RNF213 MEFs, protein expression was induced with 1 μg/mL doxycycline for at least 15 h prior to infection. Cells were infected using an MOI of 100 as described above for *Burkholderia*. At the indicated time point, cells were washed twice in PBS, fixed using 3% paraformaldehyde (PFA) for 15 min at room temperature and incubated in a quenching solution (50 mM NH_4_Cl) for 10 min. Cells were then permeabilized in 0.1% Triton X-100-PBS and incubated with appropriate primary and secondary antibodies (with DAPI) for 1 h. Samples were then mounted onto glass slides using Aqua-Poly/Mount (Polysciences, Inc.) and visualised using a confocal laser scanning microscope (LSM 710, Carl Zeiss) equipped with a Plan Apochromat 63x (Carl Zeiss) oil-immersion objective. Images were analysed with ImageJ. For scoring, at least 100 individual bacteria were blind scored from duplicate coverslips by at least two independent scorers.

### In vitro DUB assay

Following the infection of cells as described above, bacterial pellets, containing ubiquitylated-LPS, were washed and resuspended in 50 mM Tris (pH 7.4), 50 mM NaCl, 5 mM DTT and treated with the indicated purified DUB, diluted in 25 mM Tris (pH 7.4), 150 mM NaCl, 10 mM DTT at 0.5 μM +/− 10 mM Iodoacetamide for 30 min at 37 °C. Bacterial pellets were then lysed in Bugbuster and analysed by immunoblot as described above.

### Protein expression and purification

*E. coli* Rosetta cells carrying petM30-His-GST-TssM^BpΔN191^ and its variants were grown at 37 °C until OD_600_ reached 0.6, and protein expression was induced with the addition of 0.5 mM IPTG at 16 °C overnight. Cells were harvested and pellets were resuspended in 25 mM Tris-HCl (pH 7.5), 500 mM NaCl, 10 mM imidazole, 10% glycerol and lysed on ice by sonication. Lysates were clarified by centrifugation at 45,000 × *g* for 1 h, and the supernatant was applied to Ni-NTA resin for affinity purification. After binding, the resin was washed with 25 mM Tris-HCl (pH 7.5), 500 mM NaCl, 35 mM imidazole, 10% glycerol. His-GST-TssM^BpΔN191^ proteins were eluted using 25 mM Tris (pH 7.5), 250 mM NaCl, 250 mM imidazole, 10% glycerol. The His-GST tag was removed from the TssM proteins by incubating with Tobacco Etch Virus (TEV) protease for 4 h at room temperature. While cleaving, protein mixtures were dialyzed against 4 L of 25 mM Tris-HCl (pH 7.5), 250 mM NaCl, 5% glycerol to remove imidazole. After 4 hours, the solution was purified further using Ni-NTA resin pre-washed with dialysis buffer. The flowthrough was collected and concentrated for size exclusion chromatography using a Superdex 200 16/600 gel filtration column pre-equilibrated in 25 mM Tris-HCl (pH 7.4), 150 mM NaCl, 2 mM β-mercaptoethanol.

His-3C-TssM^Cs^ (59–347) was expressed from the pOPINB vector using the same induction protocol as His-GST-TssM^BpΔN191^. Purification of TssM^Cs^ was as for His-GST-TssM^BpΔN191^ with the exception of His-tag removal by 3C protease instead of TEV. All other details including buffers and columns were the same.

His-tagged human JOSD1 was expressed from a pET28b vector (kind gift from S. Buhrlage) in *E. coli* Rosetta cells. Protein expression was induced at OD_600_ of 0.6 using 0.5 mM IPTG overnight at 16 °C. The cells were harvested, resuspended, lysed and initially purified as described above for TssM. Once eluted off the Ni-NTA resin, the protein solution was concentrated for size exclusion chromatography using the same buffer as TssM^BpΔN191^.

For all proteins, protein purity was confirmed by SDS-PAGE before fractions were concentrated, quantified by absorbance, and flash frozen for storage at −80 °C. All other DUBs used in this study were purified as described previously: RickCE 378–691, SseL 24–340, ShiCE 2–405^44^; SpvD R161G full length^43^; Crimean-Congo Haemorrhagic Fever Virus vOTU 1–185^31^; LotA full length^45^, ChlaDUB1 130–401 and ChlaDUB2 80–339^48^.

### Synthesis of Rho-S(Ub)G and Rho-T(Ub)G ester-linked substrates

C-terminal tert-butyl protected dipeptides H-Ser-Gly-OtBu and H-Thr-Gly-OtBu were made using standard peptide coupling conditions. *N*,*N*’-diBoc-5-carboxy-Rhodamine^49^ was coupled to the N-terminus of both peptides, followed by esterification of the Ser and Thr sidechain OH with FmocGlyOH using EDC and HOBt. The Fmoc protecting group was removed from the Gly amine, which was subsequently coupled to fully protected Ub 1–75 with a free C-terminal carboxylic acid (prepared by solid-phase peptide synthesis^50^). Global deprotection was achieved with 90% TFA and the resulting ester-linked ubiquitin FP reagents were purified by RP-HPLC. Details are available in the Supplemental methods section.

### Fluorescence polarization (FP) deubiquitylase assays

FP was monitored using a BMG Labtech CLARIOstar microplate reader. Reaction volumes were 20 μL and data were collected in a black, low-protein binding 384-well plate at 22 °C, blanked against fresh FP buffer containing 25 mM Hepes (pH 7.4), 150 mM NaCl, 0.1 mg/mL BSA, 5 mM β-mercaptoethanol. For the ester-linked substrates conjugated to a rhodamine110 fluorophore, 482–16 nm and 530–40 nm optic filters were used for excitation and emission, respectively. For the isopeptide-linked Tamra-K(Ub)G substrate, the Tamra fluorophore was excited at 540–20 nm and emission was monitored at 590–20 nm. In each reaction, the indicated substrate (Rho-S(Ub)G, Rho-T(Ub)G, or Tamra-K(Ub)G) was present at 50 nM. For DUB panels, 0.5 μM of each DUB was used. To find the optimal concentration of TssM^Bp^ (TssM^BpΔN191^) for each substrate, a series of dilutions were prepared and FP was measured. The optimal concentration of TssM^Bp^ was 0.4 nM for ester-linked substrates and 4 nM for the isopeptide-linked substrate. These concentrations were then used to monitor activity of the indicated point mutants. All working solutions were prepared by diluting enzyme stocks into the FP buffer (25 mM Hepes (pH 7.4), 150 mM NaCl, 0.1 mg/mL BSA, 5 mM β-mercaptoethanol).

To collect data, a 2X stock of substrate (i.e., 100 nM) was added to the microplate and monitored for 5 minutes to establish a baseline FP (~177 mP). The plate was removed from the plate reader and a 2X stock of DUB was added 1:1 to establish the final reaction conditions. All reactions were performed in triplicate, and data were processed following blank subtraction. Graphs depicting WT TssM^Bp^ against TssM^Bp^ mutants show representative datasets that were collected simultaneously. For dilution experiments used to determine the catalytic efficiency of vOTU, JOSD1, TssM^Bp^, V466R TssM^Bp^, or TssM^Cs^ the FP data corresponding to substrate cleavage was analysed by non-linear regression using a one-phase decay fit from GraphPad Prism 9. Here, the Y_0_ was set to the average mP of each substrate prior to DUB addition (~177 mP), and the plateau was set to the average mP of the cleaved fluorescent peptide (~50–60 mP). For each substrate and DUB combination, the plateau was calculated from the highest concentration of DUB tested to ensure the mP value reflected a reaction that had gone to completion. Rate constants (k) were determined for each concentration by constraining k to be shared between all replicates (n=3). Rate constants and the associated standard error of the mean (SEM) were extracted and graphed against the concentration of DUB. Repeating this processing over a range of concentrations yielded linear fits that corresponded to the k_cat_/K_M_ (in M^−1^s^−1^; catalytic efficiency).

### TssM crystallization and structure determination

TssM (TssM^BpΔN191^) protein for crystallographic studies was prepared as described above, with minor changes. Following purification on Ni-NTA resin, the protein was further purified on glutathione resin prior to on-column cleavage with TEV. Ni-NTA resin was used to capture TEV protease from the eluted TssM prior to concentration and final purification by size exclusion chromatography on a Superdex 75 16/600 column. Ub-Propargylamide (PA) activity-based probe was prepared as previously described using intein chemistry^45,51^. TssM was reacted with Ub-PA at a 1:2 molar ratio overnight on a roller at room temperature in the presence of fresh 5 mM DTT. The reaction was purified by sequential rounds of ion exchange using a HiTrap Q HP column. The first round was performed in 25 mM Tris (pH 7.0), 50 mM NaCl with an elution gradient to 1 M NaCl. The TssM-Ub complex was present primarily in the flowthrough and early elution fractions, which were pooled and run over the column again at pH 8.0. The resulting TssM-Ub product was dialyzed against 20 mM Tris (pH 8.0), 150 mM NaCl and concentrated to 15 mg/mL for crystallography studies. Crystals were obtained in sitting drop format using a 100 nL drop comprised of 15 mg/mL TssM-Ub combined 1:1 with reservoir solution containing 1.4 M Sodium phosphate monobasic monohydrate/Potassium phosphate dibasic pH 5.6. Crystal trays were stored at room temperature, where crystals grew within two weeks. The resulting crystals were cryoprotected in mother liquor containing 30% glycerol prior to vitrification.

Diffraction data were collected at Diamond Light Source, Beamline I24, with a wavelength of 0.999903 Å and temperature of 100 K. The data were integrated using Dials^52^ and scaled with Aimless^53^. Molecular replacement was performed with the Phaser module of CCP4i2 using an AlphaFold model of the TssM USP domain and a previously determined structure of Ub^40,54–56^. Automated model building of the Big5 domain was performed using Buccaneer^57^, followed by iterative manual building in Coot and refinement in PHENIX^58,59^. Final Ramachandran statistics: 96.36% favored, 3.49% allowed, 0.15% outliers. All structure figures were produced using PyMol (www.pymol.org).

### Quantification and statistical analysis

Data were tested for statistical significance with GraphPad Prism software. The number of replicates for each experiment and the statistical test performed are indicated in the figure legends.

### Data and code availability

Coordinates and structure factors for the TssM-Ub structure have been deposited in the Protein Data Bank under accession code 8SSI and will be publicly available from the date of publication.

## Extended Data

**Extended Data Table 1:** Data collection and refinement statistics

	TssM-Ub
**Data collection**	
Space group	P 41 21 2
Cell dimensions	
*a*, *b*, *c* (Å)	104.52, 104.52, 193.80
α, β, γ (°)	90, 90, 90
Resolution (Å)	58.76 – 2.50 (2.60–2.50) *
*R*_merge_	0.045 (0.393)
*I* / *σI*	8.9 (1.2)
Completeness (%)	100.0 (100.0)
Redundancy	1.9 (1.9)
**Refinement**	
Resolution (Å)	52.26 – 2.50
No. reflections	37914
*R*_work_ / *R*_free_	0.2270 / 0.2536
No. atoms	
Protein	5269
Ligand/ion	14
Water	38
*B*-factors	
Protein	72.22
Ligand/ion	74.57
Water	64.29
R.m.s. deviations	
Bond lengths (Å)	0.003
Bond angles (°)	0.60

* Values in parentheses are for highest-resolution shell.
